# Ancestry, *ACKR1* and leucopenia in patients with systemic lupus erythematosus

**DOI:** 10.1136/lupus-2022-000790

**Published:** 2022-11-14

**Authors:** Cecilia P Chung, Gul Karakoc, Ge Liu, Jorge L Gamboa, Jonathan D Mosley, Nancy J Cox, C Michael Stein, Vivian Kawai

**Affiliations:** 1 Division of Rheumatology, Department of Medicine, Vanderbilt University Medical Center, Nashville, Tennessee, USA; 2 Tennessee Valley Healthcare System - Nashville Campus, Nashville, Tennessee, USA; 3 Division of Clinical Pharmacology, Department of Medicine, Vanderbilt University Medical Center, Nashville, Tennessee, USA; 4 Department of Biomedical Informatics, Vanderbilt University School of Medicine, Nashville, Tennessee, USA; 5 Vanderbilt Genetics Institute, Vanderbilt University School of Medicine, Nashville, Tennessee, USA

**Keywords:** Systemic Lupus Erythematosus, Polymorphism, Genetic, Epidemiology

## Abstract

**Objective:**

SLE is more prevalent in populations of African (AA) than European ancestry (EA) and leucopenia is common. A homozygous variant in *ACKR1* (rs2814778-CC) is associated with lower white cell counts; the variant is common in AA but not EA populations. We hypothesised that in SLE: (1) leucopenia is more frequent in patients of AA than EA, and (2) the *ACKR1-*CC genotype accounts for the higher frequency of leucopenia in AA patients.

**Methods:**

We performed a retrospective cohort study in patients with SLE at a tertiary care system. Ancestry was defined by genetic principal components. We compared the rate of leucopenia, thrombocytopenia and anaemia between (a) EA and AA patients, and (b) *ACKR1*-CT/TT and CC genotype in AA patients.

**Results:**

The cohort included 574 patients of EA and 190 of AA; *ACKR1*-CC genotype was common in AA (70%) but not EA (0%) patients. Rates of leucopenia for ancestry and genotype were AA 60.0% vs EA 36.8 % (p=1.9E-08); CC 67.7% vs CT/TT 42.1% (p=9.8E-04). The rate of leucopenia did not differ by ancestry comparing EA patients versus AA with CT/TT genotype (p=0.59). Thrombocytopenia (22.2% vs 13.2%, p=0.004) and anaemia (88.4% vs 66.2%, p=3.7E-09) were more frequent in AA patients but were not associated with *ACKR1* genotype (p=0.82 and p=0.84, respectively).

**Conclusions:**

SLE of AA had higher rates of anaemia, leucopenia, and thrombocytopenia than those of EA; only the difference in leucopenia was explained by *ACKR1*-CC genotype. This genotype could affect clinical practice.

**WHAT IS ALREADY KNOWN ON THIS TOPIC**

- Benign neutropenia due to a genetic variant in the *ACKR1* gene (rs2814778) is common in people of African ancestry and is thought to have no direct clinical consequences. However, we have previously shown that patients with this variant are more likely to have therapy with azathioprine discontinued for leucopenia. Leucopenia is more common in patients with SLE of African ancestry than those of European ancestry, but the role of the *ACKR1* genetic variation is not known and previous studies have not examined the association or the role of *ACKR1* in SLE diagnosis or treatment.

**WHAT THIS STUDY ADDS**

- This paper adds novel and clinically important data on the role of *ACKR1* genetic variation explaining lower white cell counts among patients with SLE of African compared with European ancestry. This observation is particularly important because white cell counts are used to guide cytotoxic drug therapy initiation and discontinuation.

**HOW THIS STUDY MIGHT AFFECT RESEARCH, PRACTICE OR POLICY**

- Our data raise concerns about the potential role of *ACKR1* as a factor contributing to worse outcomes among patients with SLE of African ancestry.

## Introduction

SLE is a multisystem autoimmune disease that occurs more frequently and is more severe in certain racial groups, particularly in some populations of African ancestry (AA).^1 2^ A low white cell count (leucopenia) affects approximately 50% of patients with SLE,^3^ often correlates with active disease, and is one of the haematological criteria used for disease classification.^4 5^ White cell counts are lower in patients with SLE of AA than those of European ancestry (EA),^6^ but the underlying reasons are unclear.

In the general population, individuals of AA have lower average white cell counts than those of EA and are more likely to have counts below the lower limit of laboratory reference ranges.^7^ This clinical observation had been termed ‘benign ethnic neutropenia’ and is largely attributable to high rates of homozygosity for the Duffy null red blood cell polymorphism (single nucleotide polymorphism (SNP) rs2814778) at chromosome 1q23.2 that strongly associates with lower white cell counts.^8^ The rs2814778-C SNP is in the promoter region of the human atypical chemokine receptor 1 gene (*ACKR1*) and is linked to resistance to *Plasmodium vivax* malaria and has an allele frequency that differs markedly by ancestry (96% in AA populations from west Africa, 80% in people of AA in the southwest USA and 0.6% in people of EA).^8–11^

Despite the observation that white cell counts are lower in patients with SLE of AA than those of EA, there is little information about the role of the rs2814778-CC genotype. The lower average white cell counts in patients with SLE of AA could be due to predisposing genetic factors such as *ACKR1*-CC genotype, or other factors. Because the *ACKR1* rs2814778-CC genotype does not affect platelet and red cell counts, these values could provide measures of the broad haematological effects of disease activity and cytotoxic therapy independent of genotype. Thus, we examined the hypotheses that: (1) white cell count, haemoglobin (Hb) and platelet counts are lower in AA than EA patients with SLE, and (2) that the *ACKR1* rs2814778-CC genotype accounts for the lower white cell counts but not for differences in platelet count and Hb concentration in patients of AA.

## Patients and methods

### Study population

The study population was derived from the Vanderbilt University Medical Center biobank that is linked to the de-identified electronic health record (EHR) system.^12^ We included patients with SLE; a case was defined as a patient who was diagnosed as having SLE by a specialist (rheumatologist, nephrologist or dermatologist) and the diagnosis confirmed on chart review by the investigators. This is a validated approach to identifying patients with SLE in EHRs and is the gold-standard definition used in such studies.^13 14^ To identify as many potential cases of SLE as possible, we deployed published algorithms that incorporate several clinical factors including the results for antinuclear antibody testing, billing codes, and use of medications such as hydroxychloroquine and immunosuppressants,^14^ and then reviewed EHRs of potential cases to determine true case status. The study included all patients with SLE with genotype and laboratory data available.

### Genotype data

We extracted genotype information for rs2814778, which was directly genotyped on the Illumina Infinium Multi-Ethnic Genotyping Array platform. Genotyping was performed by the Vanderbilt Technologies for Advance Genomics. Quality control was performed in PLINK V.1.90β3 following standard procedures, which include reconciling strand flips, removal of SNPs with a call rate <0.95 and samples with: (a) per-individual call rate <0.95; (b) wrong assigned sex; (c) duplicate pairs (PI-HAT ≥0.95); (d) relatedness (proportion identity by descent PI-HAT ≥0.25); (e) compromise DNA samples; and (f) SNPs that departure from Hardy Weinberg Equilibrium (HWE) (p<1×10^−6^) were also removed.^15^ Principal components (PCs), calculated using SNPRelate,^16^ were used in conjunction with HapMap populations to define ancestry by including any subject within ±4 SD of the median values for European and African recent ancestry populations.

### Haematological laboratory tests

All hemoglobin (Hb), white cell counts, and platelet counts after the first diagnosis code for SLE were extracted from structured data tables in the EHRs. For each of the selected haematological tests, the lowest and the median values were obtained. Anaemia was defined as Hb <120 g/L in females and Hb <135 g/L in males.^17^ As categorised in the SLE classification criteria,^4 5 18^ leucopenia was defined as a white cell count <4x10^9^ cells/L, neutropenia as neutrophil counts <1.5 x10^9^ cells/L and thrombocytopenia as a platelet count <100x10^9^ cells/L.

### Statistical analysis

Demographic and clinical characteristics were stratified by ancestry and rs2814778 genotype (CC vs CT/TT); the CT and TT genotypes were grouped because many studies have shown that effects on white cell count are not observed in heterozygous individuals.^8 19^ Continuous variables are presented as median (IQR) and categorical variables as frequency (percentage). Comparisons among EA and AA patients, and among rs2814778-CC and CT/TT genotypes were performed using Wilcoxon rank-sum and Χ^2^ tests for continuous and categorical variables, respectively, unless otherwise specified. A logistic regression model was performed to test the association between ancestry and leucopenia, and between leucopenia and rs2814778-CC genotype and results shown as OR and 95% CI. The following covariates were included in logistic regression analyses: median age in the EHR, age at SLE diagnosis, length of follow-up in the EHR after SLE diagnosis, and five PCs. A p value of ≤0.05 was considered significant and all analyses were performed in R v.4.1.2.

## Results

### Study population

There were 764 patients with SLE in the study, and of these, 24.9% (n=190) were of AA. Compared with those of EA, there were more females among AA patients, they were also younger, diagnosed earlier and had shorter follow-up (table 1). In addition, AA patients were more likely to have a positive antibody results and lupus nephritis (table 1). However, there were no differences for these characteristics by rs2814778 genotype (table 1). Mycophenolate, cyclophosphamide, and rituximab were more common among patients of AA compared with EA, but there were no differences for these medications by rs2814778 genotype.

**Table 1:** Characteristics of the study population by ancestry and *ACKR1* genotype (rs2814778)

Characteristics	All SLE (n=764)			Patients with SLE of AA (n=190)		
	EA (n=574)	AA (190)	P value*	*ACKR1*-CT/TT (n=57)	*ACKR1*-CC (n=133)	P value*
Age	49.6 (38.0–61.4)	38.8 (25.6–52.4)	5.4E-11	41.2 (27.1–56.3)	38.4 (25.4–51.2)	0.34
Female	502 (87.5%)	177 (93.2%)	0.03	54 (94.7%)	123 (92.5%)	0.80
Age at SLE diagnosis	44.0 (32.0–56.0)	34.0 (22.0–46.0)	4.0E-10	36.0 (23.0–48.0)	33.0 (22.0–46.0)	0.50
Lupus nephritis	99 (17.2%)	79 (41.6%)	6.2E-12	28 (49.1%)	51 (38.3%)	0.17
Positive ANA test	476 (88.6%)	160 (94.7%)	0.02	49 (94.2%)	111 (94.9%)	0.99*
Positive anti-dsDNA	239 (45.4%)	103 (58.9%)	0.002	33 (63.5%)	70 (56.9%)	0.42
Low C3 (<82 mg/dL)	152 (30.8%)	63 (40.1%)	0.03	16 (35.6%)	47 (42.0%)	0.46
Low C4 (<15 mg/dL)	174 (36.2%)	64 (42.4%)	0.17	19 (43.2%)	45 (42.1%)	0.90
Length of follow-up	7.3 (3.6–11.3)	5.0 (2.5–8.8)	8.4E-5	6.1 (2.5–10.4)	4.7 (2.5–8.4)	0.47
Hydroxychloroquine	512 (89.2%)	162 (85.2%)	0.15	52 (91.2%)	110 (82.7%)	0.20
Azathioprine	158 (27.5%)	52 (27.4%)	0.97	14 (24.6%)	38 (28.6%)	0.57
Mycophenolate	138 (24.0%)	82 (43.2%)	4.5E-7	23 (40.4%)	59 (44.4%)	0.61
Cyclophosphamide	64 (11.1%)	32 (16.8%)	0.04	6 (10.5%)	26 (19.5%)	0.13
Ciclosporin	11 (1.9%)	9 (4.7%)	0.07*	4 (7.0%)	5 (3.8%)	0.55*
Rituximab	26 (4.5%)	16 (8.4%)	0.04	6 (10.5%)	10 (7.5%)	0.69*
Belimumab	28 (4.9%)	12 (6.3%)	0.44	2 (3.5%)	10 (7.5%)	0.47*

Data are shown as median (IQR) or number (per cent).

Antinuclear antobodies (ANA) results were not available in 37 EA and 21 AA (16 with *ACKR1*-CC); anti-dsDNA in 47 EA and 15 AA (10 with *ACKR1*-CC); C3 levels in 81 EA and 33 for AA (21 with *ACKR1*-CC); C4 levels for 93 EA and 39 AA (26 with *ACKR1*-CC).

*Comparisons were performed with Pearson Χ^2^ or Fisher’s exact test for categorical, and Wilcoxon sum-rank test for continuous variables.

AA, African ancestry; anti-dsDNA, anti-double-strand DNA; EA, European ancestry.

### Effect of ancestry

**Figure 1:**
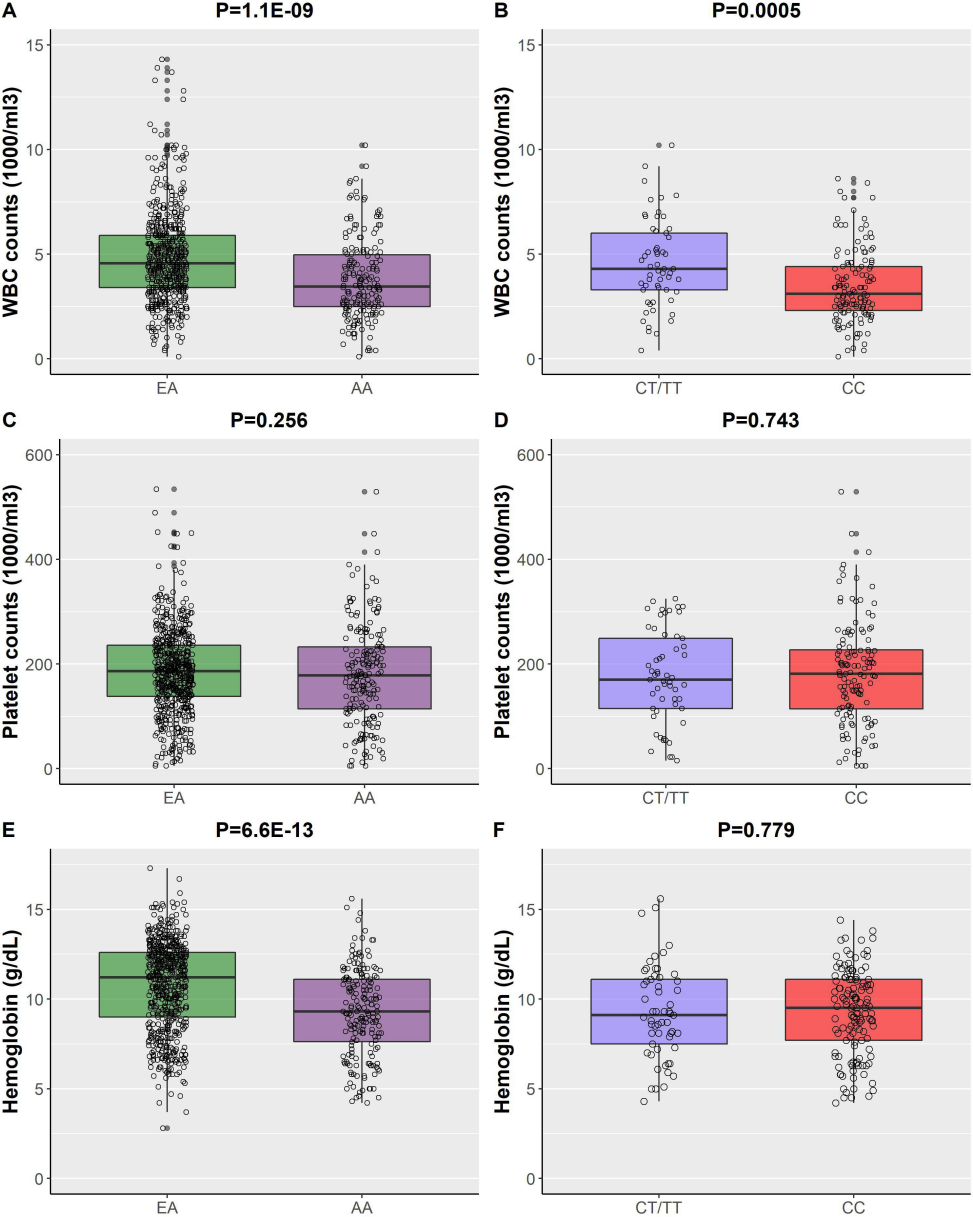
Comparisons of the lowest white blood cell (WBC) count levels (A, B), lowest platelet counts (C, D) and lowest haemoglobin levels (E, F) by ancestry and *ACKR1* genotype. Figures on the right show the comparisons between European ancestry (EA) and African ancestry (AA). Figures on the left show the comparisons between rs2814778-CT/TT and CC genotype in patients with SLE of AA.

**Figure 2:**
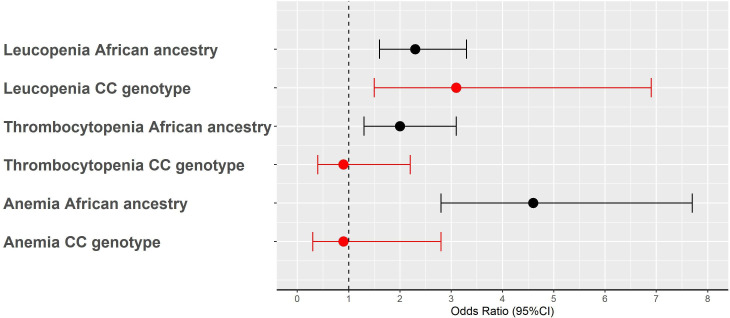
Risk of leucopenia, thrombocytopenia and anaemia in patients with SLE of African ancestry (AA) compared with European ancestry; and in carriers of rs2814778-CC genotype compared with non-carriers (CT/TT) among patients with SLE of AA. Black and red-filled dots and error bars represent the associations by ancestry and by rs2814778-CC genotype, respectively. Analyses were adjusted by sex, age, age at SLE diagnosis, length of follow-up after diagnosis of SLE and five principal components. Dots represent the OR and error bars the 95% CI.

Patients of AA had lower median and lowest levels of white cell count, neutrophil count and Hb, but not platelet count, compared with those of EA (table 2 and figure 1). AA patients were more likely to have ever had anaemia, leucopenia, neutropenia and thrombocytopenia than EA patients (p<0.005, table 2); the prevalence for leucopenia was higher in AA patients compared with EA patients (OR: 2.3, 95% CI: 1.6 to 3.3; p=6.5E-6) as was the prevalence of neutropenia (OR: 2.9, 95% CI: 1.9 to 4.5, p=1.7E-6), anaemia (OR: 4.6, 95% CI: 2.8 to 7.7; p=2.1E-9) and thrombocytopenia (OR: 2.0, 95% CI: 1.3 to 3.1; p=0.003) (figure 2).

**Table 2:** Associations of ancestry and *ACKR1* genotype (rs2814778) with haemoglobin, white cell and platelet counts in patients with SLE

Haematological parameters	All patients with SLE (n=764)			AA patients with SLE (n=190)		
	EA n=574	AA n=190	P value*	*ACKR1*-CT/TT n=57	*ACKR1*-CC n=133	P value†
Median haemoglobin (g/dL)	12.9 (11.8–13.7)	11.5 (10.4–12.5)	2.2E-16	11.8 (10.5–12.6)	11.4 (10.4–12.5)	0.53
Lowest haemoglobin (g/dL)	11.2 (9.0–12.6)	9.3 (7.6–11.1)	6.6E-13	9.1 (7.5–11.1)	9.5 (7.7–11.1)	0.78
Ever anaemia (%)	380 (66.2)	168 (88.4)	3.7E-9	50 (87.7)	118 (88.7)	0.84
Median white cell counts (10^3^/mm^3^)	6.7 (5.4–8.2)	5.9 (4.7–7.1)	2.1E-6	6.8 (5.6–8.4)	5.5 (4.4–6.7)	1.1E-4
Lowest white cell counts (10^3^/mm^3^)	4.6 (3.4–5.9)	3.5 (2.5–5.0)	1.1E-9	4.3 (3.3–6.0)	3.1 (2.3–4.4)	5.0E-4
Ever leucopenia (%)	211 (36.8)	114 (60.0)	1.9E-8	24 (42.1)	90 (67.7)	9.8E-4
Median neutrophil counts (10^3^/mm^3^)‡	4.4 (3.3–5.6)	3.7 (2.6–4.9)	3.3E-6	4.3 (3.8–4.8)	3.2 (2.4–4.4)	2.2E-5
Lowest neutrophil counts (10^3^/mm^3^)‡	2.8 (2.0–3.2)	2.0 (1.2–2.4)	2.5E-9	2.7 (1.9–3.8)	1.8 (1.1–2.7)	2.9E-4
Ever neutropenia (%)‡	58 (11.6)	61 (34.7)	6.4E-12	8 (15.1)	53 (43.1)	3.4E-8
Median platelet count (10^3^/mm^3^)	245 (201–291)	250 (202–314)	0.13	257 (202–307)	247 (202–315)	0.88
Lowest platelet count (10^3^/mm^3^)	187 (138–236)	178 (114–233)	0.26	170 (115–249)	181 (114–227)	0.74
Ever thrombocytopenic (%)	76 (13.2)	42 (22.2)	0.004	12 (21.1)	30 (22.6)	0.82

Data shown as median (IQR) or number (per cent).

Leucopenia defined as a white cell count <4000/mm^3^; neutropenia as neutrophil count <1500/mm^3^; thrombocytopenia as platelet count <100 000/mm^3^; and anaemia as haemoglobin <12 g/dL in females and Hb <13.5 g/dL in males.

*P value for EA versus AA.

†P value for CC versus CT/TT genotype in AA patients with SLE.

‡Neutrophil counts were not available in 77 patients of EA and in 14 of AA (10 with *ACKR1*-CC).

AA, African ancestry; EA, European ancestry.

### Effect of *ACKR1* rs2814778

Among patients of EA, six were heterozygous (CT) for rs2814778 and none had the CC genotype. Among AA patients, the allele frequency of the rs2814778 variant was 83.0% and was in HWE (p=0.68) with a genotype distribution as follows: TT in 6 (3.2%), CT in 51 (26.8%) and CC in 133 (70.0%) patients. In patients of AA, the median and the lowest white cell and neutrophil counts were lower in carriers of the CC genotype compared with non-carriers (p≤0.001 for all, table 2 and figure 1B), but no differences were found for the median and lowest platelet counts and Hb levels (p>0.05, table 2 and figure 1D and F). In addition, carriers of the CC genotype had higher odds of leucopenia (OR: 3.1, 95% CI: 1.5 to 6.9; p=0.003) and neutropenia (OR: 6.2, 95% CI: 2.3 to 1.9, p=0.0006) compared with non-carriers; however, the CC genotype was not associated with increased odds of thrombocytopenia (OR: 0.9, 95% CI: 0.4 to 2.2; p=0.84) or anaemia (OR: 0.9, 95% CI: 0.3 to 2.8; p=0.87) (figure 2).

The prevalence of leucopenia when comparing patients of EA and AA was no longer significant after adjustment for genotype (OR: 0.67, 95% CI: 0.26 to 1.71, p=0.41). Furthermore, when individuals with CC genotype were excluded, there were no differences in the prevalence of leucopenia between EA (33 of 396, 8.3%) and AA patients (24 of 235, 10.2%) (p=0.59).

## Discussion

The major findings of this study are that (1) patients with SLE of AA had lower white cell counts and higher prevalence of leucopenia than patients of EA; and (2) the rs2814778-CC genotype was associated with lower white cell counts in patients with SLE of AA and explained most of the observed difference between AA and EA patients. In contrast, the higher prevalence of anaemia and thrombocytopenia in patients of AA was not explained by the rs2814778-CC genotype. While the observation that the *ACKR1* rs2814778-CC genotype is associated with leucopenia in the general population is established, its contribution to leucopenia in patients with SLE has not been studied previously.

In addition to *ACKR1*-CC genotype, white cell counts in patients with SLE could have been affected by factors such as drugs, disease activity or other conditions; therefore, the lower white cell counts in AA than EA patients could plausibly be attributed to such factors. Indeed, anaemia and thrombocytopenia were also more frequent in patients of AA than EA. However, arguing against the likelihood that drugs or disease activity explained the lower white cell counts in patients of AA was the observation that the prevalence of leucopenia in EA versus AA patients was not significantly different after adjustment for genotype. Moreover, in an analysis that excluded individuals with the CC genotype, there were no significant differences in the prevalence of leucopenia between patients of EA and AA.

*ACKR1* encodes a transmembrane glycoprotein that is a receptor for pro-inflammatory cytokines and malaria parasites.^7^ The recessive allele for rs2814778, which is a point mutation in the promoter region, results in a lack of expression of ACKR1 protein on red blood cells^20^ and thus a red blood cell membrane antigen phenotype termed Duffy null or Fy(a-b-). In the absence of Duffy antigens, the malaria parasite (*P. vivax*) attaches to the red blood cells but cannot invade them.^7 21^ ACKR1 is thought to affect haematopoiesis, and the lack of ACKR1 on bone marrow erythroid cells induced the development of phenotypically distinct neutrophils that readily leave the circulation to migrate to the spleen.^22^

Carriers of the Duffy null (CC) genotype have lower neutrophil counts than non-carriers but do not have an increased risk of infection; in a UK Biobank study the CC genotype was strongly associated with lower average white cell counts but not with increased risk of viral or bacterial infection.^23^ These findings are concordant with observations that carriers and non-carriers of the CC genotype had similar inflammatory response;^24^ and differentially expressed genes were related to haematopoietic stem cell mobilisation and leucocyte migration, which may result in the migration of activated neutrophils to the spleen.^22^

Although the CC genotype is not associated with increased risk of infection or other illnesses, lower white cell counts and more frequent occurrences of leucopenia can have medical consequences that result from altered care triggered by the finding of a low white cell count.^25^ For example, individuals with the CC genotype were more likely to undergo a bone marrow biopsy for isolated leucopenia than those with CT/TT genotypes, and 97% of their biopsies had normal results.^26^

In patients with SLE, leucocyte counts are usually obtained before initiation of cytotoxic drugs, and low white cell counts could preclude patients from receiving such drugs, or such patients might receive lower doses of cytotoxic drugs or be more likely to discontinue therapy because the low white cell counts are attributed to drug toxicity.^20 27^ Further, therapeutic changes made as a result of this attributed toxicity could contribute to the poor outcomes observed more frequently among patients with SLE of AA.

In keeping with this possibility, we have recently shown that patients of AA with inflammatory diseases (inflammatory bowel disease 21%, SLE 25%, other rheumatic diseases 54%) receiving azathioprine were more likely to discontinue therapy because of leucopenia than patients of EA.^28^ Moreover, the presence of the CC genotype rather than race was the major explanation for the increased rate of azathioprine discontinuation.^28^ Also, children with lymphoma treated with 6-mercaptopurine (6-MP) were more likely to receive lower intensity doses of 6-MP if they carried the CC genotype.^28^

This study should be interpreted in the light of some potential limitations. First, haematological measurements in patients with SLE can be affected by factors related (eg, drugs, disease activity) and unrelated (eg, malignancies, bleeding) to lupus and these are likely to contribute to the differences observed in the prevalence of leucopenia, thrombocytopenia, and anaemia among patients of EA and AA. Notably, despite the higher prevalence of leucopenia in patients with the rs2814778-CC genotype, there were no differences in red blood cell or platelet indices compared with those with the CT/TT genotypes. Thus, it is likely that the difference in white cell counts between these genotype groups was largely due to the genetic variant and not to drug therapy, lupus disease activity or other factors affecting blood counts. Second, data were extracted from the EHRs of a tertiary care centre; therefore, there are potential limitations to the generalisability of our findings. Third, data completeness can be a concern with EHRs, as patients may receive care at different institutions. However, this is less likely in the setting of chronic complex diseases such as SLE.

Finally, there are several unanswered questions beyond the scope of this study. Future research will need to define whether different thresholds of leucopenia are associated with risk of infection in patients with SLE with and without the rs2814778-CC genotype, and if so, whether different white cell count thresholds should be used to guide clinical decisions about drug initiation, discontinuation or reduction of dose.

In conclusion, patients with SLE of AA had lower white cell counts and more frequent leucopenia, anaemia, and thrombocytopenia than patients of EA. Differences in white cell count measures among ancestry groups were largely explained by the rs2814778-CC genotype. Because presence of this genotype could indirectly affect decisions about cytotoxic drug initiation and dose regulation, it could potentially contribute to disparities in clinical practice and outcomes.

## Data Availability

Data are available upon reasonable request. All data and materials used in the analysis are available upon request to the corresponding author, in accordance with the funders and institutional guidance and legal requirements.
